# Examining of the mechanism by which Yin Huang Ge compound alleviates cognitive dysfunction in Alzheimer’s disease mice through modulation of Aβ degrading enzymes and neurotrophic factors

**DOI:** 10.3389/fnagi.2026.1821074

**Published:** 2026-06-17

**Authors:** Yaxuan Hao, Haoyue Yang, Jianmin Zhong, Jiayun Zhang, Xiaoping He, Xianhui Dong

**Affiliations:** Key Laboratory of Traditional Chinese Medicine for the Prevention and Treatment of Cardiovascular and Cerebrovascular Diseases, Hebei University of Chinese Medicine, Shijiazhuang, Hebei, China

**Keywords:** Alzheimer disease, Aβ deposition, cognitive impairment, neurotrophic factors, Yin Huang Ge compound

## Abstract

**Background:**

Alzheimer’s disease (AD) poses a significant threat to human health, and with the number of patients increasing annually, developing effective prevention and treatment strategies has become an urgent priority. Early intervention is a viable strategy for treating AD, as biomarker changes associated with *β*-amyloid (Aβ) can emerge 20 or more years before cognitive impairment becomes apparent. Traditional Chinese medicine (TCM) offers a potential avenue for treatment at this early stage.

**Objective:**

This study investigated the potential therapeutic effects of the Yin Huang Ge compound on cognitive function in a mouse model of AD and evaluated its efficacy in mitigating Aβ accumulation, a key pathological feature of AD.

**Methods:**

APP/PS1 mice were randomly assigned to the following groups: Controls (C57BL/6 J mice) group, the Model group, YHG group, and donepezil (DNP) group. The YHG group and the DNP group were administered their respective drugs through gastric gavage, whereas the normal group and the Model group were supplied with saline. Administration sustained for a period of 30 consecutive days. We assessed mouse learning, memory, and spatial cognition using the Morris water maze, novel object recognition, Y-maze spontaneous alternation, and novel arm tests. Western blotting quantified the protein levels of *β*-amyloid 1–42 (Aβ_1-42_), insulin-degrading enzyme (IDE), neprilysin (NEP), matrix metalloproteinase-2 (MMP-2), and matrix metalloproteinase-9 (MMP-9). ELISA measured IDE and NEP enzymatic activity, while immunohistochemistry evaluated the expression of brain-derived neurotrophic factor (BDNF) and neurotrophin-3 (NT-3).

**Results:**

In contrast to the Control group, mice in the Model group exhibited significant impairments in learning, memory, and spatial cognition. The protein expression levels of Aβ_1-42_, MMP-2, and MMP-9 were markedly elevated (*p* < 0.01), whereas the expression of IDE and NEP was significantly reduced (*p* < 0.05, *p* < 0.01). ELISA analysis further revealed a substantial decrease in IDE and NEP activity (*p* < 0.01). A concomitant reduction in the expression of NT-3 and BDNF was also observed (*p* < 0.01). Compared to the Model group, mice treated with YHG or DNP exhibited significantly improved learning, memory, and spatial cognition. The protein expression of Aβ_1-42_, MMP-2, and MMP-9 was reduced (*p* < 0.05, *p* < 0.01), whereas the expression of IDE and NEP was elevated (*p* < 0.05). ELISA results confirmed the increased activity of IDE and NEP (*p* < 0.05). These assays also revealed a marked upregulation in the expression of NT-3 and BDNF (*p* < 0.01).

**Conclusion:**

The YHG compound reduces cerebral Aβ deposition via a bidirectional regulatory mechanism, upregulating IDE and NEP to promote clearance while downregulating MMP-2 and MMP-9 to mitigate associated damage. It also elevates the expression of the neurotrophic factors BDNF and NT-3, which enhances endogenous neuroprotection and ameliorates core Alzheimer’s disease pathology. Consequently, this treatment markedly improves cognitive function in APP/PS1 mice.

## 1 Introduction

Alzheimer’s disease (AD) is a progressive neurological disorder characterized by a gradual onset (Abubakar et al., 2022; Tahami Monfared et al., 2022). Clinical symptoms typically worsen over time, with the initial phase characterized by memory impairment and cognitive decline. Consequently, patients develop verbal communication difficulties, psychological and behavioral abnormalities, and a restricted range of outcomes, ultimately progressing to total disability or mortality (Tarawneh and Holtzman, 2012). Worldwide, the global population of individuals with dementia is projected to increase from 55 million in 2019 to 139 million by 2050 (Chen et al., 2024). The imbalance between Aβ synthesis and clearance is a primary driver in the earliest stages of AD. This dysregulation leads to the formation of Aβ plaques, which are neurotoxic (Azargoonjahromi, 2024; Cavallucci et al., 2012). This neurotoxicity induces excessive phosphorylation of the microtubule-associated protein tau, triggers neuroinflammation, and causes neuronal death, ultimately leading to cognitive impairment (Liang et al., 2022). However, contemporary pharmacological treatments for AD, including cholinesterase inhibitors and amyloid-targeted agents, can alleviate certain symptoms but are associated with a range of adverse effects.(Miculas et al., 2022). Consequently, developing effective pharmaceuticals for the prevention of AD has become increasingly vital (Cao et al., 2018). Traditional Chinese medicine (TCM) employs a multi-targeted approach with minimal adverse effects, which demonstrates significant potential for applications such as treating AD (Liu et al., 2025). The primary pathogenesis of AD is fundamentally rooted in a deficiency of kidney essence and an insufficiency of the marrow sea. Phlegm-stasis obstructing the orifices represents a secondary manifestation (Zhao et al., 2022). The Yin Huang Ge compound (YHG), which contains icariin (Jin et al., 2019), astragaloside IV (Abd Elrahim Abd Elkader et al., 2022), and puerarin (Abd Elrahim Abd Elkader et al., 2022), was developed based on the etiology and pathogenesis of AD and the TCM principle of holistically addressing both root causes and symptoms. This compound contains active constituents derived from the herbs Epimedium, Astragalus, and Pueraria (Zhang et al., 2024). The YHG compound revitalizes the spleen and kidneys, replenishes essence and marrow, nourishes cerebral marrow, clears the orifices, alleviates muscular tension, and stimulates the meridians. It improves the circulation of qi and blood, thereby effectively alleviating AD symptoms. The YHG compound improves renal function, replenishes essence and marrow, nourishes the cortical bone, clears the orifices, relieves muscle tension, activates the collaterals, and promotes the circulation of qi and blood (Dong et al., 2015). This YHG compound has significantly alleviated symptoms of atopic dermatitis. The research team’s prior findings indicated that the YHG biological exhibits multi-target preventive and therapeutic effects in APP/PS1 mice, including anti-inflammatory, antioxidant, and neuroprotective properties (Yu et al., 2019). The study involved APP/PS1 double transgenic mice, with DNP utilized as the positive control medication. This examination of the correlation between Aβ clearance and cognitive impairments in AD model mice elucidates the therapeutic potential of the YHG compound, providing additional evidence to bolster its advancement for the prevention and mitigation of AD.

## 2 Materials

### 2.1 Experimental animals

Twenty-four male SPF-grade APP/PS1 double transgenic mice, aged 4 months and weighing (25 ± 5) g, were used, together with six male C57BL/6 J mice of the same age. All mice were obtained from Beijing Huafukang Biotechnology Co., Ltd. (certificate number: NO.11032225110173075). The Animal Ethics Committee of Hebei University of Chinese Medicine approved all experimental procedures (approval number: KJLL(D)20,251,003). The animals were maintained under a 12-h light/dark cycle at 20–25 °C and 40–70% humidity. All mice were housed individually.

### 2.2 Study design and blinding implementation

All behavioral and biochemical experiments in this study were conducted under strict blinding conditions. Grouping information was coded and sealed by researchers not involved in subsequent experiments, and staff responsible for animal feeding, drug administration and behavioral testing only knew the animal codes throughout the process, with no knowledge of strain allocation and grouping information. The operation and analysis of Morris water maze, Y maze and novel object recognition tests, as well as the detection and statistical analysis of Western Blot, ELISA and immunohistochemistry, were all independently completed by different researchers in a blinded state, and unblinding was only performed after all experiments were completed and data were locked.

### 2.3 Pharmaceutical chemicals

Icariin, astragaloside IV, and puerarin (A0145, A0070, A0068) were obtained from Chengdu Manster Biotechnology Co., Ltd. with a purity of ≥98%. DNP hydrochloride (1D2120) was sourced from Beijing Solarbio Technology Co., Ltd. Rabbit anti-neprilysin (NEP), rabbit anti-insulin-degrading enzyme (IDE), polyclonal anti-Aβ_1-42_ antibody, and rabbit anti-matrix metalloproteinase-2 (MMP-2) antibody were supplied by Chengdu Zhengneng Biotechnology Co., Ltd. (batch numbers: R381344, 381594, R23330 and R380817). Antibodies against β-actin and matrix metalloproteinase-9 (MMP-9) (lot numbers 20536-1-AP and 27306-1-AP) were purchased from Wuhan Sanying Technology Co., Ltd. ECL (AP34L044) was obtained from Heyuan Li Ji Biological Company. IDE and NEP ELISA Kits (USEB897Mu, USEB785Mu) were acquired from Wuhan Yunke Long Technology Co., Ltd.

## 3 Methods

### 3.1 Animal administration and groupings

APP/PS1 transgenic mice were randomly assigned to the Model group, the YHG treatment group, or the DNP treatment group, with six mice per group. Six age-matched C57BL/6 J mice served as the Control group. An orthogonal trial was conducted to determine the appropriate dosage of the Epimedium-Astragalus-Pueraria mixture, which contained 120 mg/kg icariin, 80 mg/kg astragaloside IV, and 80 mg/kg puerarin (Xian-Hui et al., 2021). For drug preparation, the three monomers were individually dissolved in normal saline, diluted to the required concentration, and mixed immediately prior to oral gavage. The DNP treatment group received 3.25 mg/kg DNP daily via oral administration. The control and experimental groups were given an equal volume of distilled water orally once daily for 30 days.

### 3.2 Morris water maze experiment

Following the YHG intervention, an adaptive training phase was conducted, which was subsequently validated by a six-day Morris water maze experiment. The circular pool was divided into four quadrants, one of which contained a submerged escape platform. Each mouse received two training trials per day at fixed intervals. The escape latency, defined as the time required for a mouse to locate the submerged platform within 60 s of entry, was recorded for each trial. On the final day, the platform was removed for a spatial probe test. Each mouse was placed into the pool and allowed to swim freely. During the 120-s test, the number of crossings over the former platform location and the time spent in the target quadrant were recorded.

### 3.3 Y maze experiment

The Y-maze apparatus comprises three arms, each 45 cm long, 12 cm high, and 5 cm wide at the passage. These arms are designated 1, 2, and 3. A mouse was gently restrained and then placed at the maze’s central intersection, facing a predetermined direction. The animal was allowed to explore the arms freely for 5 min, during which the sequence and total number of arm entries were recorded. An entry into three different arms in consecutive order was counted as one correct alternation. The spontaneous alternation rate was calculated as [Number of correct alternations / (Total arm entries - 2)] × 100%.

### 3.4 New object recognition experiment

Day 1 serves as the acclimatization phase, during which no objects are placed in the open-field arena. On Day 2, two identical cylindrical objects, designated A and B, are positioned 5 cm from the adjacent walls. The mouse is introduced to the arena with its back to the objects and allowed to explore them for 5 min; exploration times for objects A and B are recorded. The test phase begins on Day 3, when object B is replaced with a novel cubic object, designated C, in the same location. The mouse is again placed with its back to the objects and permitted 5 min of exploration, with times for objects A and C documented. The recognition index is calculated as (exploration time for the novel object) / (exploration time for the novel object + exploration time for the familiar object) × 100%. After each test, feces are collected and the arena is wiped with ethanol to eliminate residual odors.

### 3.5 Western blot assessment of the expression levels of Aβ associated with clearance proteins Aβ_1-42_, IDE, NEP, MMP-2, and MMP-9

Proteins were isolated from murine cerebral tissue using a lysis buffer, and the supernatant protein concentration was determined via the BCA assay. The proteins were separated by SDS-PAGE and transferred to a polyvinylidene fluoride (PVDF) membrane. The membrane was then blocked with 5% skim milk for 2 h at room temperature. Following three 10-min TBST washes, the membrane was incubated with the primary antibody overnight at 4 °C with gentle shaking. It was then incubated with the appropriate secondary antibody. Protein bands were detected using an ECL chemiluminescence system, with β-actin as the loading control. Band intensities were quantified using Quantity One software. The gray value of each target protein band was normalized to that of the β-actin band from the same lane. For each experiment, the normalized ratios from three independent biological replicates (*n* = 3) were averaged. Relative protein expression levels are presented as the mean optical density ratio (target/β-actin) ± SEM.

### 3.6 ELISA the examination of IDE and NEP activity

Blood was collected via the mouse orbital sinus technique. Prior to collection, the whiskers were trimmed and the eyeball was enucleated using curved forceps. The blood was collected into a 1.5 mL centrifuge tube and allowed to clot at room temperature for 30 min. It was then centrifuged at 4 °C and 3,000 rpm for 15 min. The supernatant was obtained, labeled with the sample identifier, and stored at −80 °C for subsequent analysis. For the ELISA, we used mouse IDE and NEP ELISA kits (SEB897Mu, Cloud-Clone Corp., China), which have a minimum detectable dose of ≤0.055 ng/mL and an assay range of 0.156-10 ng/mL. The intra-assay coefficient of variation (CV) was <10%, the inter-assay CV was <12%, and activity loss during the validity period was controlled within 5%. All antibody systems in the kit were pre-optimized and ready for use without further dilution. The biological activity of IDE and NEP in the murine central nervous system was assessed strictly according to the ELISA kit protocol.

### 3.7 Immunohistochemical comprehension of neurotrophic factor NT-3 and BDNF expression levels

During fixation in 4% paraformaldehyde for 24 h, the brain tissue was dehydrated, sterilized, and embedded in paraffin before sectioning. The paraffin sections were dewaxed in heated water and subjected to antigen retrieval with EDTA buffer. They were then incubated in a 3% hydrogen peroxide solution at 37 °C for 25 min. After three washes with PBS buffer, the sections were blotted dry and covered uniformly with a drop of 3% BSA for 30 s. A secondary antibody was applied and incubated at room temperature for 50 min. The DAB substrate was used for development, and the reaction was stopped by rinsing with tap water. Counterstaining was performed with hematoxylin, followed by dehydration, clearing, and mounting. The sections were examined under a microscope for imaging. For each section, five non-overlapping fields were randomly imaged at 200 × magnification. Using ImageJ software, the integrated optical density (IOD) of DAB-positive staining was measured within a manually outlined target area. Relative protein expression was calculated as the ratio of IOD to the total measured area. Three consecutive sections per animal were analyzed, and the average value was used for subsequent statistical analysis.

### 3.8 Statistical analysis

Data were analyzed using SPSS version 25.0. Quantitative data are presented as the mean±standard error of the mean (SEM). All quantitative data were first tested for normality and homogeneity of variance to satisfy the assumptions of one-way ANOVA. Data meeting these assumptions were analyzed by one-way ANOVA, with Tukey’s test for *post-hoc* comparisons, while categorical data were evaluated using chi-square tests. We also calculated the effect size (*η*^2^) and 95% confidence intervals for all primary outcomes to quantify the magnitude of treatment effects and the precision of the estimates. *p* < 0.05 was considered statistically significant.

## 4 Result

### 4.1 Efficacy of YHG on memory and spatial cognitive functions in Alzheimer’s disease model mice

#### 4.1.1 Morris water maze test results

**Figure 1:**
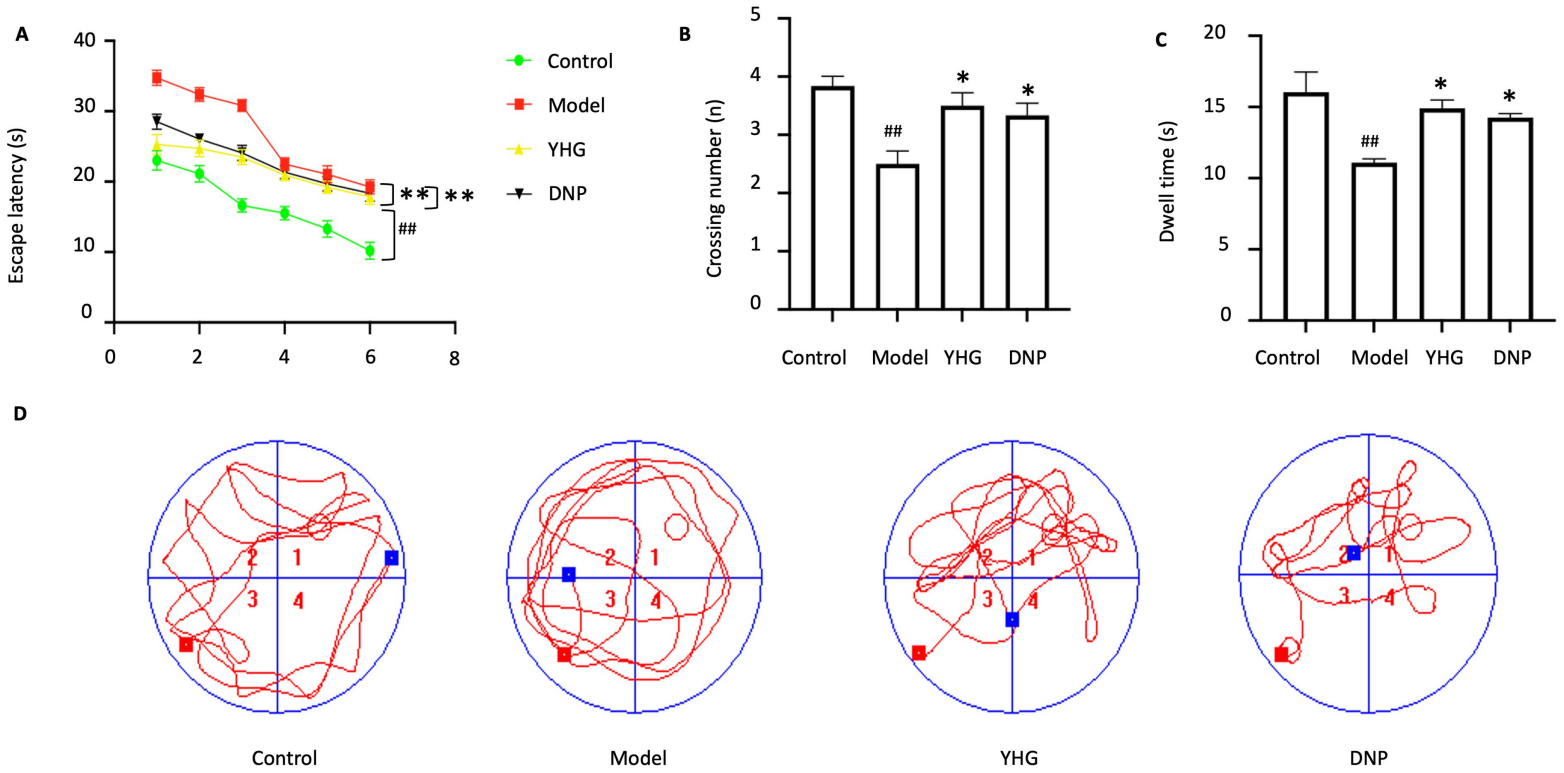
Results of Morris water maze experiment for the mice of each group. **(A)** Escape latency; **(B)** Crossing number of the platform; **(C)** Dwell time in target quadrant; **(D)** Movement traces in probe trial of Morris water maze. Data are expressed as mean ± SEM, *n* = 6. ^*^p < 0.05, ^**^p < 0.01 vs. Model group; ^#^*p* < 0.05, ^##^*p* < 0.01 vs. Control group.

Mice in the Model group exhibited a significantly prolonged latency to escape (*p* < 0.01), a substantially reduced duration in the target quadrant (*p* < 0.01), a considerable decline in platform crossings (*p* < 0.01), and erratic movement patterns compared to the Control group. In contrast to the Model group, the escape latency significantly decreased (*p* < 0.01), the total time spent in the target quadrant was markedly increased (*p* < 0.05), the average number of platform crossings was significantly rose (*p* < 0.05), and the intentionality of platform seeking was enhanced in the YHG and DNP treatment groups. Relative to the DNP treatment group, the YHG group demonstrated no statistically significant differences in escape latency, duration spent in target quadrants, or frequency of platform crossings (*p* > 0.05). The Morris water maze’s behavioral performance can be seen in Figure 1 and Table 1.

**Table 1:** Cognitive Performance in the Morris Water Maze Test.

Grouping	Probe trial target quadrant performance		*p*-value	
	Dwell time (s)	Crossing number (n)	*p*-value (Dwell time)	*p*-value (Crossing number)
Control (*n* = 6)	16.03 ± 1.43	3.83 ± 0.16		
Model (*n* = 6)	11.16 ± 0.23	2.5 ± 0.22	0.0010##	0.0015##
YHG (*n* = 6)	14.90 ± 0.57	3.50 ± 0.22	0.0137*	0.0143*
DNP (*n* = 6)	14.21 ± 0.30	3.33 ± 0.21	0.0460*	0.0497*

All data are presented as mean±SEM, with 6 mice per group. ^*^p < 0.05, ^**^p < 0.01 vs Model group; ^#^*p* < 0.05, ^##^*p* < 0.01 vs Control group. Statistical comparisons among multiple groups were performed using one-way analysis of variance (ANOVA) followed by Tukey’s *post-hoc* test. After adaptive training, the mice were subjected to 6 consecutive days of place navigation training with 2 trials per day, and the escape latency to find the submerged platform within 60 s was recorded. On the 6th day, a 120-s probe trial was performed after removing the platform, and the number of platform crossings and the time spent in the target quadrant were recorded.

#### 4.1.2 Morris water maze test results

**Figure 2:**
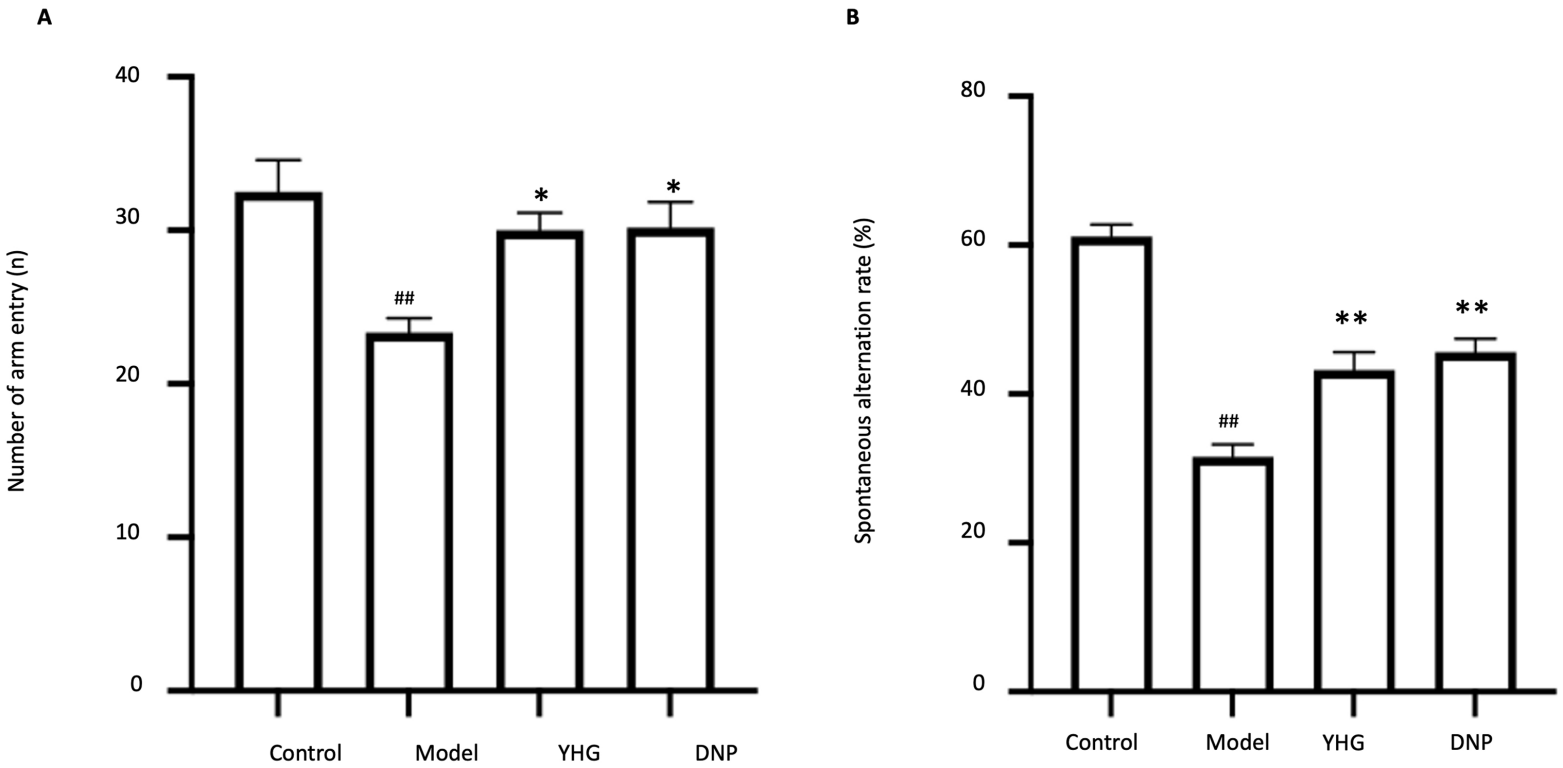
Total arm entries and spontaneous alternation rate in the Y-maze test for each group of mice. **(A)** Number of arm entries, reflecting the locomotor activity of mice; **(B)** Spontaneous alternation rate, reflecting the spatial working memory of mice. Data are expressed as mean ± SEM, *n* = 6. ^*^p < 0.05, ^**^p < 0.01 vs. Model group; ^#^*p* < 0.05, ^##^*p* < 0.01 vs. Control group.

Mice with this Model group exhibited a significantly reduced number of total arm entries (*p* < 0.01), a decreased alternation rate (*p* < 0.01), and an elevated percentage of errors compared to the Control group, indicating impaired memory function. In as compared to Model mice, both the YHG and DNP treatment groups demonstrated significantly increased total arm entries (*p* < 0.05), markedly enhanced alternation rates (*p* < 0.01), and considerably fewer errors. No statistically significant difference was observed between the YHG group and the DNP treatment group (*p* > 0.05). The outcomes of the Y-maze spontaneous alternation test are illustrated in Figure 2 and Table 2.

**Table 2:** Total arm entries and spontaneous alternation rate in y-maze test.

Grouping	Y-maze spontaneous alternation test		*P*-value	
	Total arm entries(n)	Spontaneous alternation rate (%)	P-value(Total arm entries)	P-value(Spontaneous alternation rate)
Control (*n* = 6)	32.5 ± 2.08	61.14 ± 1.61		
Model (*n* = 6)	26.17 ± 1.54	31.57 ± 1.67	0.0021^##^	0.0001^##^
YHG (*n* = 6)	29.17 ± 1.49	43.21 ± 1.42	0.0280*	0.0018**
DNP (*n* = 6)	30.17 ± 1.68	45.6 ± 1.85	0.0237*	0.0002**

Data are expressed as mean ± SEM, *n* = 6. Total arm entries indicate the locomotor activity of mice, while spontaneous alternation rate was calculated as (number of correct alternations / (total arm entries - 2)) × 100% to evaluate working memory. ^*^p < 0.05, ^**^p < 0.01 vs Model group; ^#^*p* < 0.05, ^##^*p* < 0.01 vs Control group. Statistical comparisons among multiple groups were performed using one-way analysis of variance (ANOVA) followed by Tukey’s *post-hoc* test.

**Figure 3:**
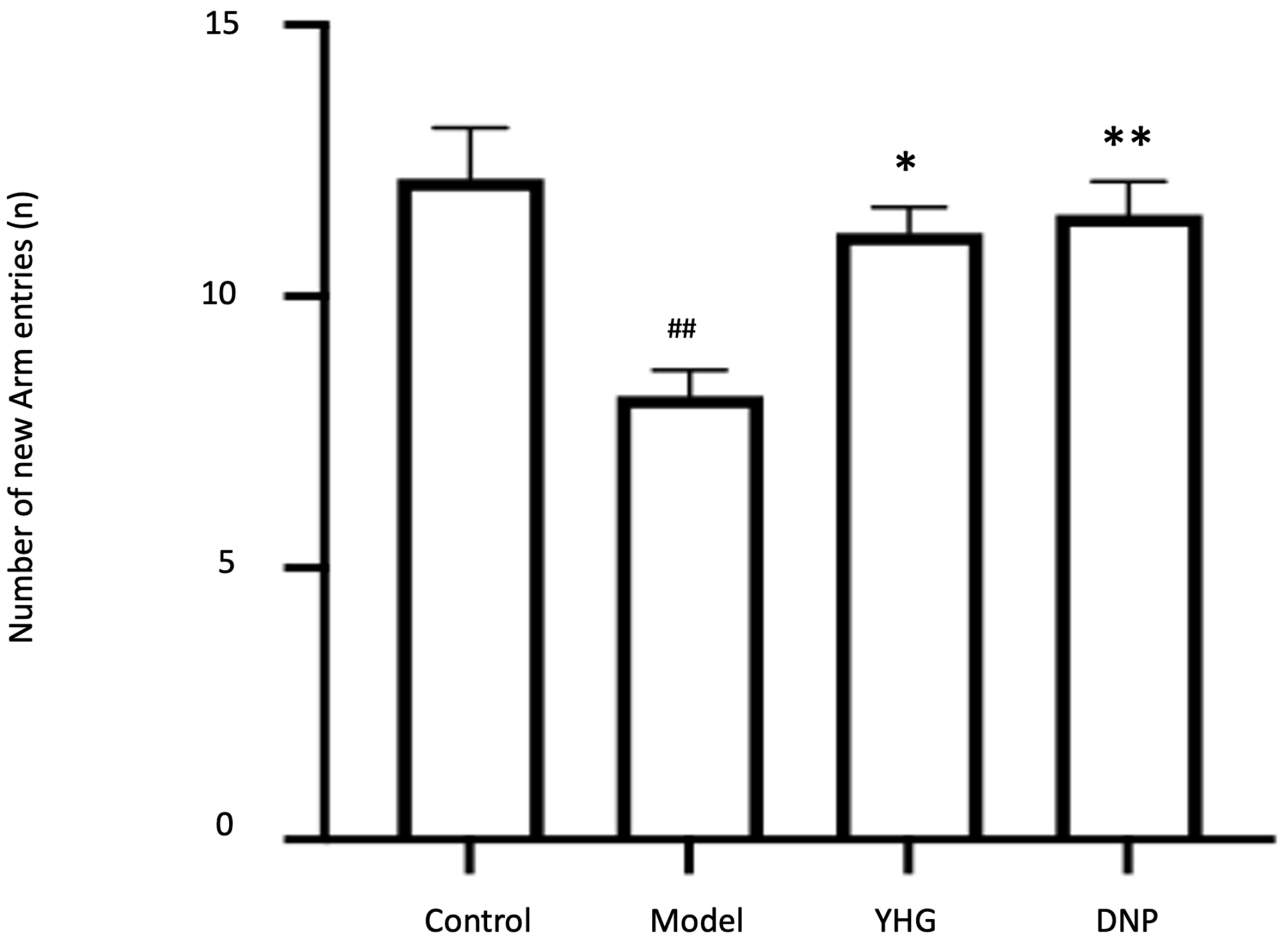
Number of entries into the novel arm during the Y-maze test for each group of mice, reflecting the ability of mice to recognize new spatial information. Data are expressed as mean ± SEM, *n* = 6. ^*^p < 0.05, ^**^p < 0.01 vs. Model group; ^#^*p* < 0.05, ^##^*p* < 0.01 vs. Control group.

Mice in the Model group exhibited a notable reduction in the frequency of crossings into the novel arm relative to the Control group (*p* < 0.01). In comparison to the AD disease model group mice, the proportion of entries in the YHG and DNP groups significantly considerably (*p* < 0.05, *p* < 0.01); However, no statistically significant difference was observed between YHG and DNP groups (*p* > 0.05). Figure 3 and Table 3 depict the findings and conclusions of the Y-maze novel arm assessment.

**Table 3:** Number of entries into the novel arm in Y-maze test.

Grouping	Novel arm exploration	*P*-value
	Number of novel arm entries (n)	*P*-value (Number of novel arm entries)
Control (*n* = 6)	12.16 ± 0.95	
Model (*n* = 6)	8.83 ± 0.7	0.0018^##^
YHG (*n* = 6)	11 ± 0.58	0.0205*
DNP (*n* = 6)	11.33 ± 0.76	0.0093**

Data are expressed as mean ± SEM, *n* = 6. The number of novel arm entries reflects the ability of mice to recognize and explore new environments, with higher values indicating stronger recognition memory. ^*^p < 0.05, ^**^p < 0.01 vs Model group; ^#^*p* < 0.05, ^##^*p* < 0.01 vs Control group. Statistical comparisons among multiple groups were performed using one-way analysis of variance (ANOVA) followed by Tukey’s *post-hoc* test.

#### 4.1.3 Experimental outcomes for novel object investigation

**Figure 4:**
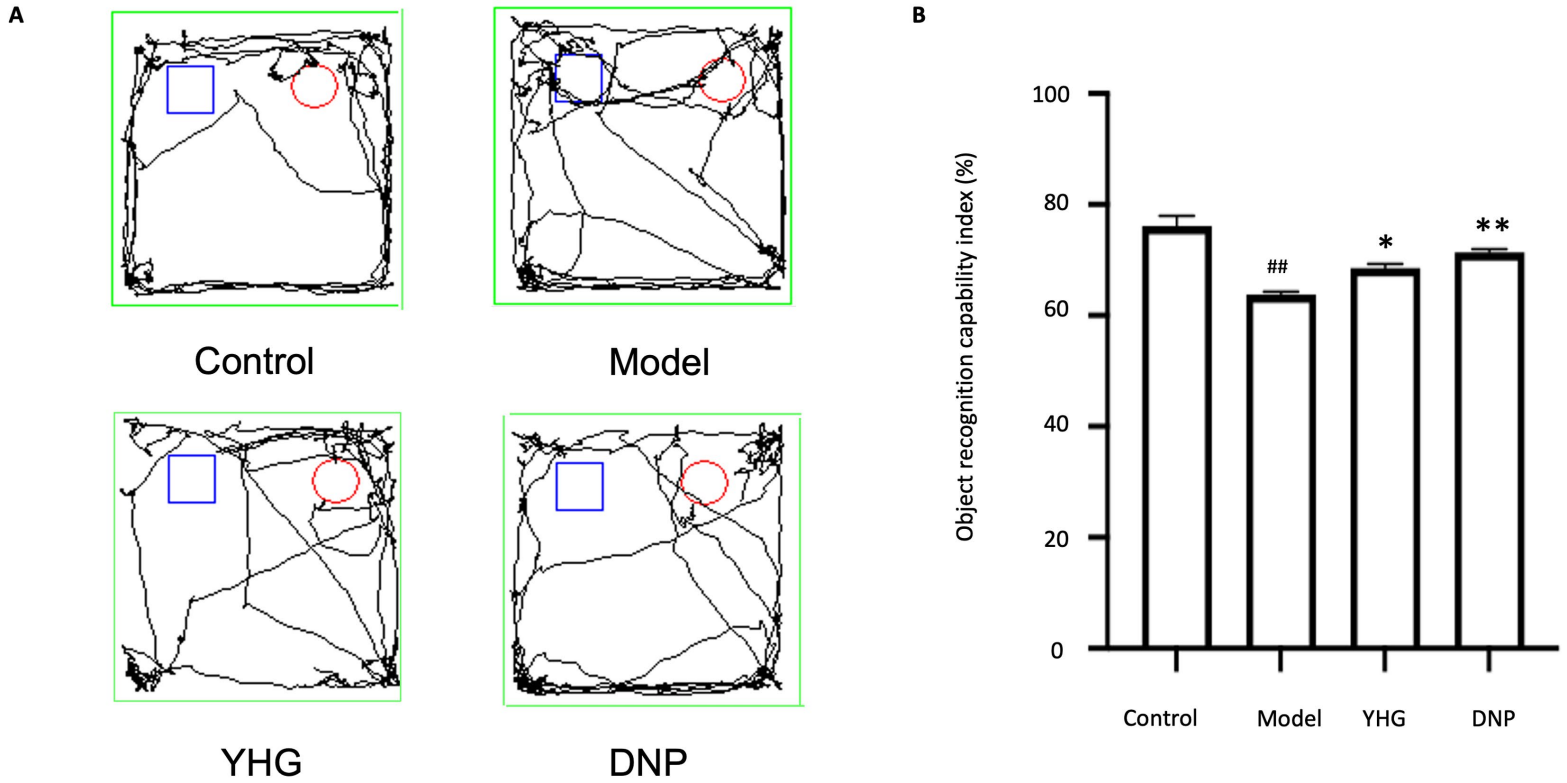
Object recognition ability index for each group of mice. **(A)** Activity Trajectory Diagram; **(B)** Object recognition index. Data are expressed as mean ± SEM, *n* = 6. ^*^p < 0.05, ^**^p < 0.01 vs. Model group; ^#^*p* < 0.05, ^##^*p* < 0.01 vs. Control group.

Compared to the Control group, mice in the Model group exhibited restricted activity ranges and similar exploratory behaviors towards both novel and familiar objects, along with a significantly reduced object recognition ability index (*p* < 0.01). Contrary to Model mice, both the YHG and DNP groups exhibited increased activity ranges and a propensity for extended duration in the central zone. Both groups demonstrated heightened exploratory behavior towards novel objects, accompanied by significantly improved object recognition ability indices (*p* < 0.05, *p* < 0.01), indicating ameliorated memory function in the mice. No statistically significant difference was detected between the YHG and DNP treatment cohorts (*p* > 0.05). Figure 4 and Table 4 illustrate the results of the novel object test.

**Table 4:** Results of novel object recognition test in mice.

Grouping	Novel object recogniton	*P*-value
	Recogniton index (%)	*P*-value (Number of novel arm entries)
Control (*n* = 6)	75.56 ± 1.52	
Model (*n* = 6)	64.20 ± 1.20	0.0001^##^
YHG (*n* = 6)	66.47 ± 1.35	0.0259^*^
DNP (*n* = 6)	72.04 ± 0.77	0.0005^**^

Data are expressed as mean ± SEM, *n* = 6. The recognition index is calculated as the percentage of time spent exploring the novel object relative to the total exploration time of both ability in mice. ^*^p < 0.05, ^**^p < 0.01 vs Model group; ^#^*p* < 0.05, ^##^*p* < 0.01 vs Control group. Statistical comparisons among multiple groups were performed using one-way analysis of variance (ANOVA) followed by Tukey’s *post-hoc* test.

### 4.2 Effects of YHG on the expression levels of Aβ clearance-related proteins Aβ_1-42_, IDE, NEP, MMP-2, and MMP-9

**Figure 5:**
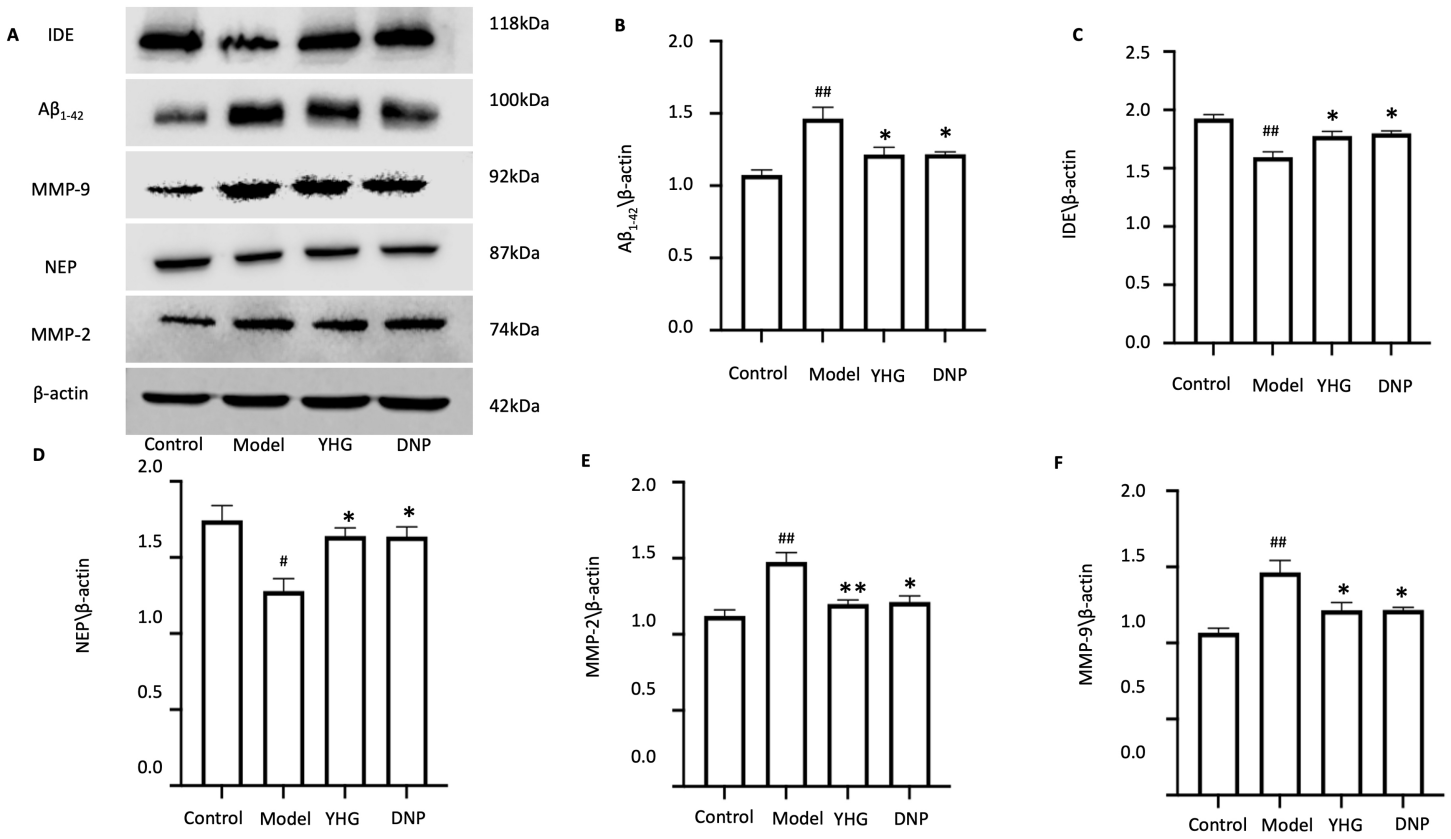
Protein expression levels of Aβ_1-42_ (amyloid-β protein 1–42), IDE (insulin-degrading enzyme), NEP (neprilysin), MMP-2 (matrix metalloproteinase-2), and MMP-9(matrix metalloproteinase-9) in each group of mice. **(A)** Electrophoresis of Aβ_1-42_, IDE, NEP, MMP-2, and MMP-9 protein; **(B)** Aβ_1-42_ protein expression compared with β-actin; **(C)** IDE protein expression compared with β-actin; **(D)** NEP protein expression compared with β-actin; **(E)** MMP-2 protein expression compared with β-actin; **(F)** MMP-9 protein expression compared with β-actin. Mean ± SEM, *n* = 6, ^*^p < 0.05, ^**^p < 0.01 vs. Model group; ^#^*p* < 0.05, ^##^*p* < 0.01 vs. Control group.

Contrary to the Control group, the Model group exhibited a significant increase in the amounts of Aβ_1-42_, MMP-2, and MMP-9 proteins (*p* < 0.01), while the expression levels of IDE and NEP were drastically lowered (*p* < 0.05, *p* < 0.01). Compared to the Model group, the protein expression levels of Aβ_1-42_, MMP-2, and MMP-9 were significantly reduced in both the YHG treatment groups and the DNP treatment group (*p* < 0.05, *p* < 0.01), while the protein expression levels of IDE and NEP increased considerably (*p* < 0.05). No significant difference was observed between the YHG and DNP treatment groups (*p* > 0.05). The data indicate that the YHG compound can increase NEP and IDE levels while inhibiting MMP-2 and MMP-9 expression, thereby improving central Aβ degradation. Figure 5 depicts the detailed protein expression levels of Aβ_1-42_, IDE, and NEP in the brains of mice across all groups.

### 4.3 Effects of YHG on IDE and NEP activity in AD model mice

**Figure 6:**
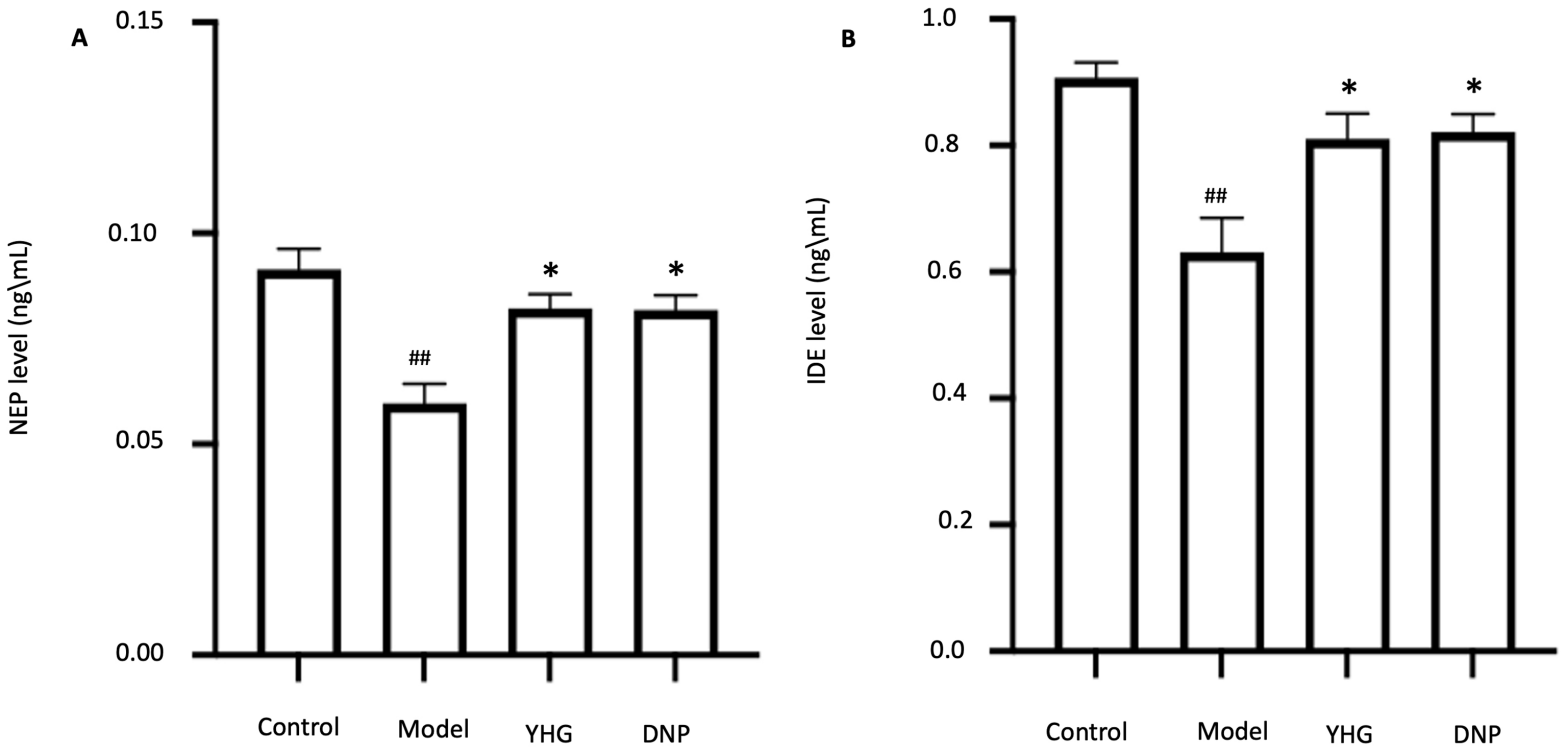
Results of ELISA experiment for each group of mice. **(A)** NEP (neprilysin) level of mice in each group; **(B)** IDE (insulin-degrading enzyme) level of mice in each group. Data are expressed as mean ± SEM, *n* = 6. ^*^p < 0.05, ^**^p < 0.01 vs. Model group; ^#^*p* < 0.05, ^##^*p* < 0.01 vs. Control group.

Contrary to the Control group, the Model group demonstrated a significant reduction in IDE and NEP activity (*p* < 0.01). In comparison to the Model group, IDE and NEP levels significantly increased in both the YHG and DNP treatment groups (*p* < 0.05). No substantial difference was observed between the YHG and DNP treatment groups (*p* > 0.05). The aforementioned results indicate that the YHG compound elevates the levels of the two chief degradation enzymes, IDE and NEP, thereby facilitating the degradation of Aβ. Comprehensive IDE and NEP activity data for each cohort of mice are showcased in Figure 6.

### 4.4 YHG effects on neurotrophic factors NT-3 and BDNF in the hippocampal region of AD model mice

**Figure 7:**
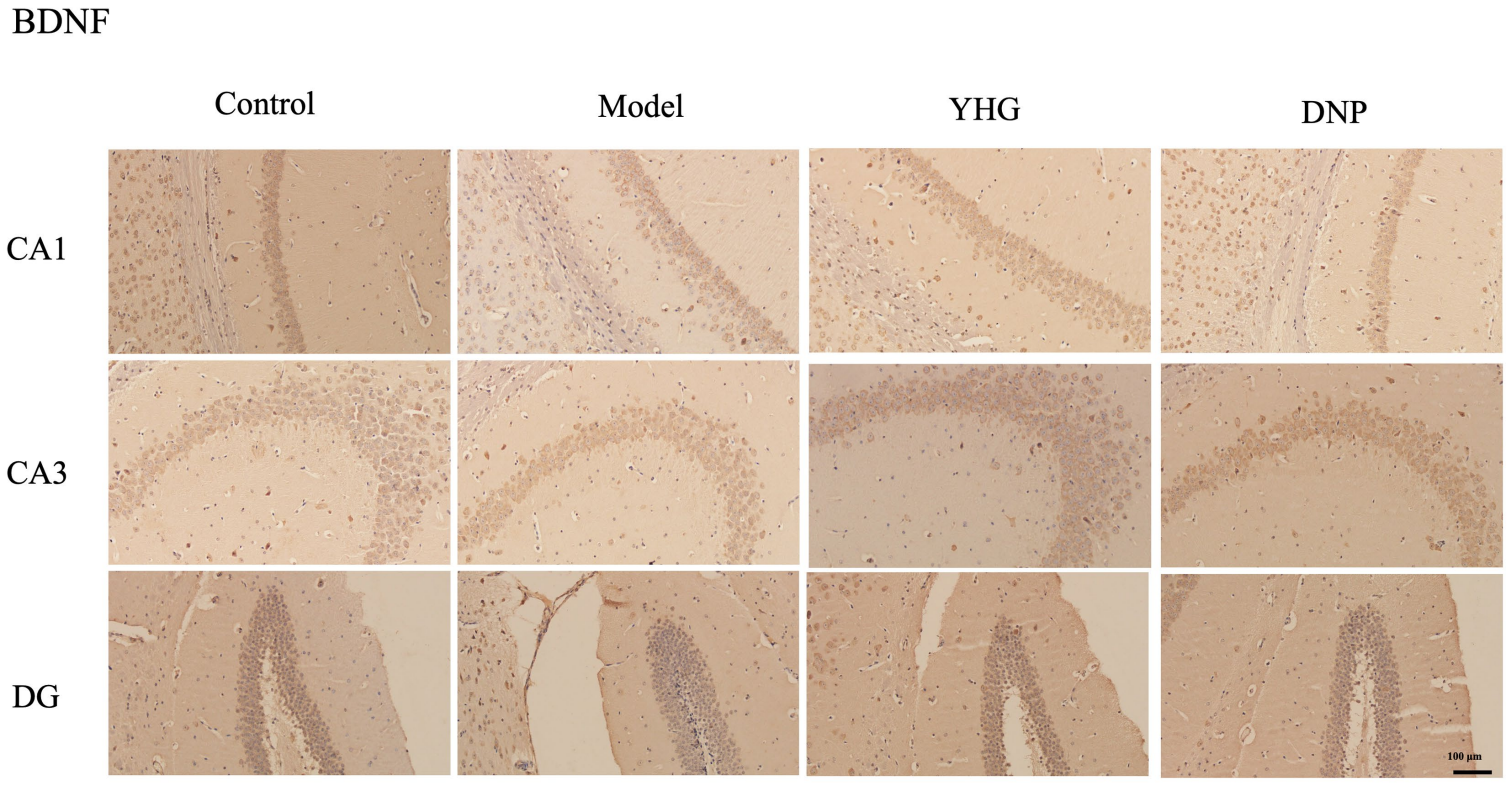
Effects on the optical density of BDNF (brain-derived neurotrophic factor) proteins in hippocampal CA1, CA3 and DG tissues of each group of mice (IHC × 100). The brownish-yellow particles indicate the positive expression of BDNF, and the darker the color, the higher the expression level of BDNF.

**Figure 8:**
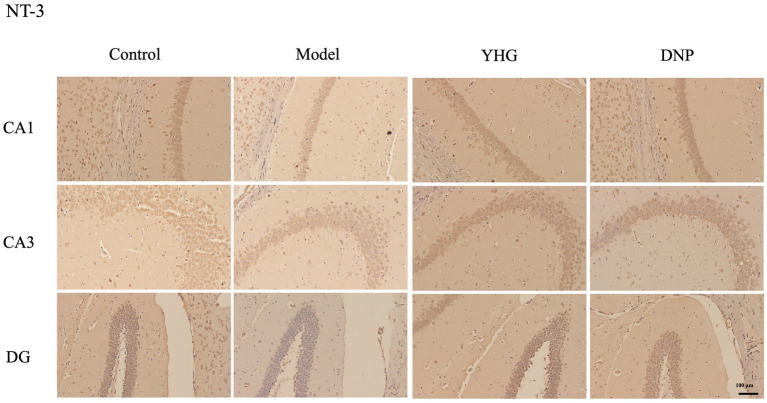
Effects on optical density of NT-3 (neurotrophin-3) proteins in hippocampal tissues in each group of mice (IHC × 100). The brownish-yellow precipitate indicates the positive expression of NT-3 protein, and the expression level is positively correlated with the color depth.

Compared to the Control group, the Model group exhibited significantly reduced levels of the neurotrophic factors NT-3 and BDNF in the hippocampal region (*p* < 0.01). According to the Model group, the treatment groups with YHG and DNP treatment groups demonstrated an increased expression of neurotrophic factors NT-3 and BDNF in the hippocampal region (*p* < 0.01); with minimal difference observed between the YHG and DNP treatment groups (*p* > 0.05). The aforementioned results indicate that the YHG compound may enhance the expression of two neurotrophic factors, NT-3 and BDNF, promote neuronal repair, and improve cognitive impairment. The immunohistochemical findings for NT-3 and BDNF in each mouse group are illustrated in Figures 7, 8, as well as in Tables 5, 6.

**Table 5:** Optical density of BDNF protein in hippocampal subregions determined by immunohistochemistry.

Grouping	Hippocampal BDNF protein expression			*P*-value		
	CA1 region optical density	CA3 region optical density	DG region optical density	*P*-value (CA1)	*P*-value (CA3)	*P*-value (DG)
Control (*n* = 6)	0.29 ± 0.0012	0.28 ± 0.0032	0.26 ± 0.0012			
Model (*n* = 6)	0.25 ± 0.0025	0.21 ± 0.0009	0.16 ± 0.0013	0.000001^##^	0.000001^##^	0.000001^##^
YHG (*n* = 6)	0.27 ± 0.0019	0.24 ± 0.0003	0.19 ± 0.012	0.000013**	0.000001**	0.000001**
DNP (*n* = 6)	0.28 ± 0.0017	0.28 ± 0.0021	0.20 ± 0.0012	0.000006**	0.000001**	0.000001**

Data are presented as mean±SEM, n = 6. The expression level of BDNF protein in the CA1, CA3 and DG subregions of the hippocampus was detected by immunohistochemical staining, and the mean optical density value was used to reflect the abundance of protein expression. ^*^p < 0.05, ^**^p < 0.01 vs Model group; ^#^*p* < 0.05, ^##^*p* < 0.01 vs Control group. Statistical comparisons among multiple groups were performed using one-way analysis of variance (ANOVA) followed by Tukey’s *post-hoc* test.

**Table 6:** Optical density of NT-3 protein in hippocampal subregions determined by immunohistochemistry.

Grouping	Hippocampal NT-3 protein expression			*P*-value		
	CA1 region optical density	CA3 region optical density	DG region optical density	*P*-value (CA1)	*P*-value (CA3)	*P*-value (DG)
Control (*n* = 6)	0.29 ± 0.007	0.29 ± 0.0017	0.28 ± 0.0020			
Model (*n* = 6)	0.21 ± 0.0021	0.21 ± 0.0019	0.20 ± 0.0022	0.000001**	0.000001**	0.000001**
YHG (*n* = 6)	0.27 ± 0.006	0.25 ± 0.0020	0.27 ± 0.0009	0.000001^##^	0.000001^##^	0.000001^##^
DNP (*n* = 6)	0.28 ± 0.0006	0.26 ± 0.0007	0.27 ± 0.0018	0.000001^##^	0.000001^##^	0.000001^##^

Data are expressed as mean ± SEM, *n* = 6. The expression level of NT-3 protein in CA1, CA3 and DG subregions of the hippocampus was detected by immunohistochemical staining, and the mean optical density value was used to reflect the abundance of its expression. Increased expression of NT-3 usually indicates enhanced neuroprotective or restorative effects. ^*^p < 0.05, ^**^p < 0.01 vs Model group; ^#^*p* < 0.05, ^##^*p* < 0.01 vs Control group. Statistical comparisons among multiple groups were performed using one-way analysis of variance (ANOVA) followed by Tukey’s *post-hoc* test.

## 5 Conclusion

Alzheimer’s disease is a neurological disorder characterized by progressive cognitive decline (Keller, 2006). The etiology is multifaceted and the pathophysiology is intricate, with a primary mechanism involving the accumulation of Aβ in the brain and a disturbance in its clearance (Penke et al., 2017). Current therapeutic agents are often limited by their single-target specificity, high cost, and propensity for adverse effects (Hossain and Hussain, 2025). TCM is a significant cultural heritage that has demonstrated unique efficacy in managing AD symptoms (Chen et al., 2023). Classical Chinese medicine theory holds that AD is characterized by a combination of deficiency and excess (Li and Yang, 2024). It originates from anomalies in qi and blood and dysfunction of the internal organs, with the brain serving as the primary site of affliction. The fundamental pathophysiology involves insufficient renal essence, which results in inadequate nourishment of the marrow sea (Tan et al., 2022). The secondary pathogenesis involves phlegm-turbidity and stasis-toxin. In this formulation, Epimedium acts as the sovereign herb to warm kidney yang, replenish essence and marrow, and enhance cognitive function (Zhuang et al., 2023). Astragalus significantly replenishes vital energy and enhances blood circulation, thereby facilitating the unblocking of meridians (Ma et al., 2025). Pueraria is employed to enhance and fortify the clear yang. This formula effectively tonifies the kidneys, replenishes essence, invigorates qi, enhances blood circulation, opens the orifices, and resolves phlegm, directly addressing the essence of AD (Wang and Wang, 2024). Behavioral assessments—the Morris water maze, novel object recognition test, and Y-maze test—demonstrated that the YHG compound significantly enhanced learning, memory, and spatial cognition in AD model mice, with effects comparable to the positive control drug DNP. The compound thus improves cognitive functions relevant to AD, providing a behavioral basis for subsequent mechanistic studies.

This study investigates Aβ metabolism at the molecular level. Discrepancies in Aβ synthesis and degradation, particularly of the more aggregation-prone Aβ_1-42_, are significant factors in neurotoxicity and plaque formation (Pauwels et al., 2012). Current research indicates that while Aβ_1-42_ constitutes only 10–20% of total Aβ, its greater propensity for aggregation and fibrillation amplifies its neurotoxicity, establishing it as the primary toxic species (Oren et al., 2021). Therefore, reducing Aβ_1-42_ levels is a critical therapeutic objective in AD. Studies have shown that the YHG compound significantly lowers Aβ_1-42_ concentrations in the brains of AD model mice. Mechanistic investigation further revealed that YHG enhances the expression and activity of two key Aβ-degrading enzymes: IDE and NEP. Western Blot and ELISA assays confirmed the upregulation of IDE and NEP activity, which correlated significantly with improved cognitive performance in behavioral tests. Extensive studies have established that diminished activity of IDE and NEP is closely linked to increased Aβ deposition and subsequent cognitive decline (Eckman and Eckman, 2005). In patients with AD and in animal models, the expression of IDE and NEP is frequently and significantly reduced. This downregulation impairs the clearance of Aβ, which in turn accelerates cognitive decline (Nalivaeva et al., 2008). Therefore, restoring or upregulating the expression of IDE and NEP is considered an effective strategy for ameliorating cognitive dysfunction in AD. Concurrently, the YHG compound also inhibited the expression of matrix metalloproteinases MMP-2 and MMP-9. These enzymes can exacerbate Aβ-associated pathological damage during AD progression by compromising blood–brain barrier integrity and intensifying neuroinflammation (Yang, 2025). Elevated levels of MMP-2 and MMP-9 in AD are associated with blood–brain barrier disruption and neuroinflammation, processes that directly contribute to cognitive decline. The inhibition of MMP-9 alleviates cognitive deficits in animal models of AD, further underscoring the detrimental role of these proteases in cognitive function (Mizoguchi et al., 2009). The YHG compound’s inhibition of MMP-2 and MMP-9 suggests a potential to enhance Aβ clearance indirectly by stabilizing the cerebral microenvironment. IDE and NEP are the principal rate-limiting enzymes for Aβ degradation within the central nervous system, and their expression levels correlate positively with Aβ clearance rates (Wang et al., 2006). The observed upregulation of IDE and NEP following YHG treatment aligns with their established biological functions, offering indirect yet compelling evidence for YHG-facilitated Aβ degradation. In contrast, MMP-2 and MMP-9 are known to exacerbate Aβ deposition and neuroinflammation in AD by compromising blood–brain barrier integrity (Wang et al., 2021);the inhibition of these MMPs by YHG further supports its role in indirectly promoting Aβ clearance by maintaining homeostasis in the cerebral microenvironment. Thus, the YHG compound may reduce Aβ accumulation and neurotoxicity via a dual mechanism: it enhances degradation by upregulating IDE and NEP while suppressing damage pathways by downregulating MMP-2 and MMP-9. This finding provides a novel perspective on its role in diminishing Aβ deposition.

Alongside Aβ elimination, protecting neurons and restoring brain function constitute another crucial therapeutic objective for AD (Folch et al., 2016). Achieving this goal requires maintaining normal neurotrophic factor function. Aβ deposition induces oxidative stress and inflammation, which disrupts this process. The progression of AD involves neuronal death or a reduction in neuron populations within memory-related brain regions, ultimately resulting in cognitive impairment (Goel et al., 2022). Neurotrophic factors are essential protein molecules that regulate neuronal development, survival, and function (Huang and Reichardt, 2001). They are essential for nervous system development, function, and neural network formation. Our findings show that the YHG compound significantly increases hippocampal expression of the neurotrophic proteins BDNF and NT-3 in a mouse model of AD. BDNF principally acts through its high-affinity receptor, tyrosine kinase receptor B (TrkB), a relationship critical for neuronal survival, synaptic plasticity, and cognitive functions such as learning and memory (Azman and Zakaria, 2022). NT-3 promotes neuronal activity and is critical for both neuronal development and the maintenance of function (Ding et al., 2025). Excessive Aβ accumulation damages neurons, in part by impairing their nutritional supply (Bayer and Wirths, 2010). The YHG compound enhances the expression of BDNF and NT-3, which likely improves the survival and functional recovery of injured neurons. This effect acts synergistically with the previously outlined Aβ clearance mechanism. Together, these processes form the molecular and cellular basis for the alleviation of cognitive impairment.

The present research demonstrates that YHG significantly improves cognitive deficits in APP/PS1 transgenic mice. The mechanism likely involves a bidirectional modulation of Aβ metabolism, promoting clearance through the upregulation of IDE and NEP while mitigating damage via the downregulation of MMP-2 and MMP-9. Furthermore, YHG enhances endogenous neuroprotection by upregulating the neurotrophic factors BDNF and NT-3. Similar neuroprotective effects are evident in other TCM formulas for AD, including He Xiaoyao Powder, and Buyang Huanwu Decoction. He Xiaoyao Powder attenuates Aβ deposition, neuroinflammation, and oxidative stress, thereby improving cognitive function in AD models (Wang et al., 2025). Buyang Huanwu Decoction also provides neuroprotection by suppressing Aβ production, protecting neurons, and maintaining blood–brain barrier integrity (Mu et al., 2014). Although our group has conducted long-term, systematic research on this compound, the evidence remains confined to animal models, and its clinical application faces considerable limitations. Translating these findings to human patients requires further validation through pharmacodynamic, pharmacokinetic, and safety studies.

## Data Availability

The raw data supporting the conclusions of this article will be made available by the authors, without undue reservation.
