# Supplementary material for: Examining of the mechanism by which Yin Huang Ge compound alleviates cognitive dysfunction in Alzheimer’s disease mice through modulation of Aβ degrading enzymes and neurotrophic factors
Source: Front Aging Neurosci. 2026 Jun 17;18:1821074. doi: 10.3389/fnagi.2026.1821074 (PMC13318864; doi:10.3389/fnagi.2026.1821074)
Supplement: Supplementary file 1 [file Data_Sheet_1.DOCX]

Supplementary Material

**Supplementary Table 1. Number of Platform Crossings and Time Spent in Target Quadrant in Morris Water Maze Test**

|  | Group | | | |
| --- | --- | --- | --- | --- |
|  | Control group | Model group | YHG group | DNP group |
| Number of platform crossings | 4 | 2 | 4 | 4 |
|  | 4 | 3 | 3 | 3 |
|  | 3 | 2 | 4 | 4 |
|  | 4 | 3 | 3 | 3 |
|  | 4 | 3 | 3 | 3 |
|  | 4 | 2 | 4 | 3 |
| Time spent in target quadrant | 13.00 | 10.85 | 16.65 | 14.50 |
|  | 14.50 | 10.20 | 13.90 | 13.90 |
|  | 22.80 | 11.25 | 13.80 | 13.90 |
|  | 16.10 | 12.20 | 14.10 | 13.80 |
|  | 15.75 | 11.05 | 14.20 | 15.50 |
|  | 14.05 | 11.03 | 16.80 | 14.25 |

**Supplementary Table 2. Escape Latency in Morris Water Maze Test**

| Group | | | | | | | | | | | | | | | | | | | | | | | |
| --- | --- | --- | --- | --- | --- | --- | --- | --- | --- | --- | --- | --- | --- | --- | --- | --- | --- | --- | --- | --- | --- | --- | --- |
| Control group  1d | Control group  2d | Control group  3d | Control group  4d | Control group  5d | Control group  6d | Model group  1d | Model group  2d | Model group  3d | Model group  4d | Model group  5d | Model group  6d | YHG group  1d | YHG group  2d | YHG group  3d | YHG group  4d | YHG group  5d | YHG group  6d | DNP group  １d | DNP group  2d | DNP group  3d | DNP group  4d | DNP group  5d | DNP group  5d |
| 25.10 | 25.40 | 15.00 | 18.20 | 12.50 | 10.40 | 29.10 | 34.20 | 29.80 | 25.00 | 24.40 | 23.60 | 28.90 | 28.10 | 25.10 | 23.10 | 23.00 | 23.00 | 28.10 | 27.90 | 26.00 | 19.80 | 19.20 | 23.50 |
| 23.10 | 17.90 | 17.80 | 14.20 | 10.20 | 15.80 | 33.80 | 33.80 | 29.00 | 23.50 | 21.30 | 17.80 | 25.60 | 27.20 | 26.10 | 19.80 | 19.80 | 16.10 | 25.10 | 26.20 | 26.10 | 25.00 | 24.00 | 16.10 |
| 18.90 | 19.80 | 15.60 | 15.10 | 17.80 | 8.60 | 35.60 | 33.00 | 28.10 | 21.80 | 17.20 | 19.40 | 20.10 | 25.50 | 25.70 | 17.80 | 17.80 | 17.20 | 27.10 | 26.10 | 25.70 | 22.00 | 20.10 | 18.00 |
| 27.80 | 18.80 | 14.00 | 12.10 | 11.30 | 9.50 | 35.00 | 31.10 | 27.50 | 22.00 | 19.20 | 17.90 | 22.30 | 20.00 | 19.30 | 18.10 | 18.10 | 17.00 | 29.00 | 25.00 | 19.30 | 21.90 | 18.00 | 17.50 |
| 19.50 | 23.80 | 16.80 | 17.60 | 15.80 | 6.90 | 34.80 | 26.50 | 23.00 | 18.00 | 18.50 | 18.00 | 28.70 | 23.50 | 22.30 | 17.20 | 17.20 | 17.80 | 33.00 | 27.10 | 23.40 | 20.40 | 19.00 | 19.00 |
| 23.60 | 20.90 | 20.50 | 15.90 | 12.00 | 9.80 | 28.00 | 30.00 | 26.80 | 20.00 | 20.10 | 15.00 | 26.00 | 24.90 | 22.30 | 19.20 | 19.20 | 15.90 | 28.70 | 24.10 | 24.00 | 18.00 | 17.00 | 15.90 |

**Supplementary Table 3. Raw Data of Spontaneous Alternation Rate, Total Arm Entries and Novel Arm Exploration in Y-Maze Test**

|  | Group | | | |
| --- | --- | --- | --- | --- |
|  | Control group | Model group | YHG group | DNP group |
| Total arm entries | 38 | 24 | 35 | 35 |
|  | 23 | 25 | 29 | 33 |
|  | 32 | 20 | 31 | 30 |
|  | 35 | 21 | 28 | 23 |
|  | 34 | 26 | 27 | 31 |
|  | 33 | 24 | 30 | 29 |
| Spontaneous alternation(%) | 62.18 | 35.68 | 40.16 | 45.19 |
|  | 65.66 | 35.23 | 43.28 | 48.48 |
|  | 58.93 | 29.35 | 45.19 | 49.25 |
|  | 60.15 | 25.13 | 33.19 | 39.17 |
|  | 55.12 | 30.16 | 49.28 | 50.21 |
|  | 64.82 | 33.89 | 48.19 | 41.34 |
| Novel arm entries | 13 | 9 | 12 | 13 |
|  | 14 | 6 | 11 | 11 |
|  | 9 | 9 | 11 | 13 |
|  | 10 | 9 | 10 | 11 |
|  | 12 | 8 | 13 | 9 |
|  | 15 | 8 | 10 | 12 |

**Supplementary Table 4. Discrimination Index in Novel Object Recognition Test**

|  | Group | | | |
| --- | --- | --- | --- | --- |
|  | Control group | Model group | YHG group | DNP group |
| Discrimination Index  (%) | 78.65 | 60.50 | 64.97 | 75.13 |
|  | 79.20 | 63.70 | 65.98 | 69.85 |
|  | 74.60 | 65.50 | 69.90 | 71.30 |
|  | 70.24 | 64.20 | 70.05 | 70.68 |
|  | 80.30 | 62.16 | 67.80 | 72.40 |
|  | 76.20 | 63.30 | 68.95 | 72.87 |

**Supplementary Table 5. Quantitative analysis of relative protein expression**

|  | Group | | | |
| --- | --- | --- | --- | --- |
|  | Control group | Model group | YHG group | DNP group |
| Aβ_1-42_\β-actin | 1.06252 | 1.5984 | 1.13900 | 1.202100 |
|  | 1.02290 | 1.3258 | 1.19830 | 1.251300 |
|  | 1.13850 | 1.4962 | 1.31040 | 1.97400 |
| NEP\β-actin | 1.875 | 1.405 | 1.508 | 1.508 |
|  | 1.803 | 1.125 | 1.704 | 1.704 |
|  | 1.552 | 1.705 | 1.698 | 1.698 |
| IDE\β-actin | 1.977 | 1.539 | 1.721 | 1.813 |
|  | 1,936 | 1.563 | 1.855 | 1.827 |
|  | 1.862 | 1.685 | 1.751 | 1.758 |
| MMP-2\β-actin | 1.120 | 1.590 | 1.150 | 1.140 |
|  | 1.050 | 1.380 | 1.210 | 1.230 |
|  | 1.190 | 1.460 | 1.240 | 1.270 |
| MMP-9\β-actin | 1.0620 | 1.5984 | 1.1390 | 1.2021 |
|  | 1.0210 | 1.3258 | 1.19830 | 1.2513 |
|  | 1.1200 | 1.4962 | 1.31040 | 1.1974 |

**Supplementary Table 6. The protein activity of IDE and NEP detected by ELISA**

|  | Group | | | |
| --- | --- | --- | --- | --- |
|  | Control group | Model group | YHG group | DNP group |
| NEP | 0.0873 | 0.0628 | 0.0790 | 0.0791 |
|  | 0.1011 | 0.0656 | 0.0785 | 0.0779 |
|  | 0.0859 | 0.0505 | 0.0889 | 0.0886 |
| IDE | 0.9537 | 0.5291 | 0.7560 | 0.7932 |
|  | 0.8719 | 0.6544 | 0.7891 | 0.7927 |
|  | 0.8945 | 0.7109 | 0.8870 | 0.8782 |

**Supplementary Table 7. Quantitative analysis of BDNF and NT3 by IOD**

|  | Group | | | |
| --- | --- | --- | --- | --- |
|  | Control group | Model group | YHG group | DNP group |
| BDNF CA1 | 0.287 | 0.241 | 0.277 | 0.274 |
|  | 0.283 | 0.248 | 0.271 | 0.279 |
|  | 0.285 | 0.249 | 0.276 | 0.279 |
| BDNF CA3 | 0.284 | 0.205 | 0.244 | 0.277 |
|  | 0.278 | 0.201 | 0.245 | 0.279 |
|  | 0.289 | 0.204 | 0.244 | 0.272 |
| BDNF DG | 0.258 | 0.155 | 0.195 | 0.195 |
|  | 0.254 | 0.157 | 0.198 | 0.192 |
|  | 0.257 | 0.159 | 0.194 | 0.191 |
| NT-3 CA1 | 0.289 | 0.232 | 0.283 | 0.282 |
|  | 0.287 | 0.235 | 0.281 | 0.280 |
|  | 0.298 | 0.237 | 0.282 | 0.281 |
| NT-3 CA3 | 0.281 | 0.209 | 0.266 | 0.262 |
|  | 0.288 | 0.202 | 0.269 | 0.266 |
|  | 0.285 | 0.203 | 0.267 | 0.268 |
| NT-3 DG | 0.281 | 0.0209 | 0.266 | 0.262 |
|  | 0.288 | 0.202 | 0.269 | 0.266 |
|  | 0.285 | 0.203 | 0.267 | 0.2 |


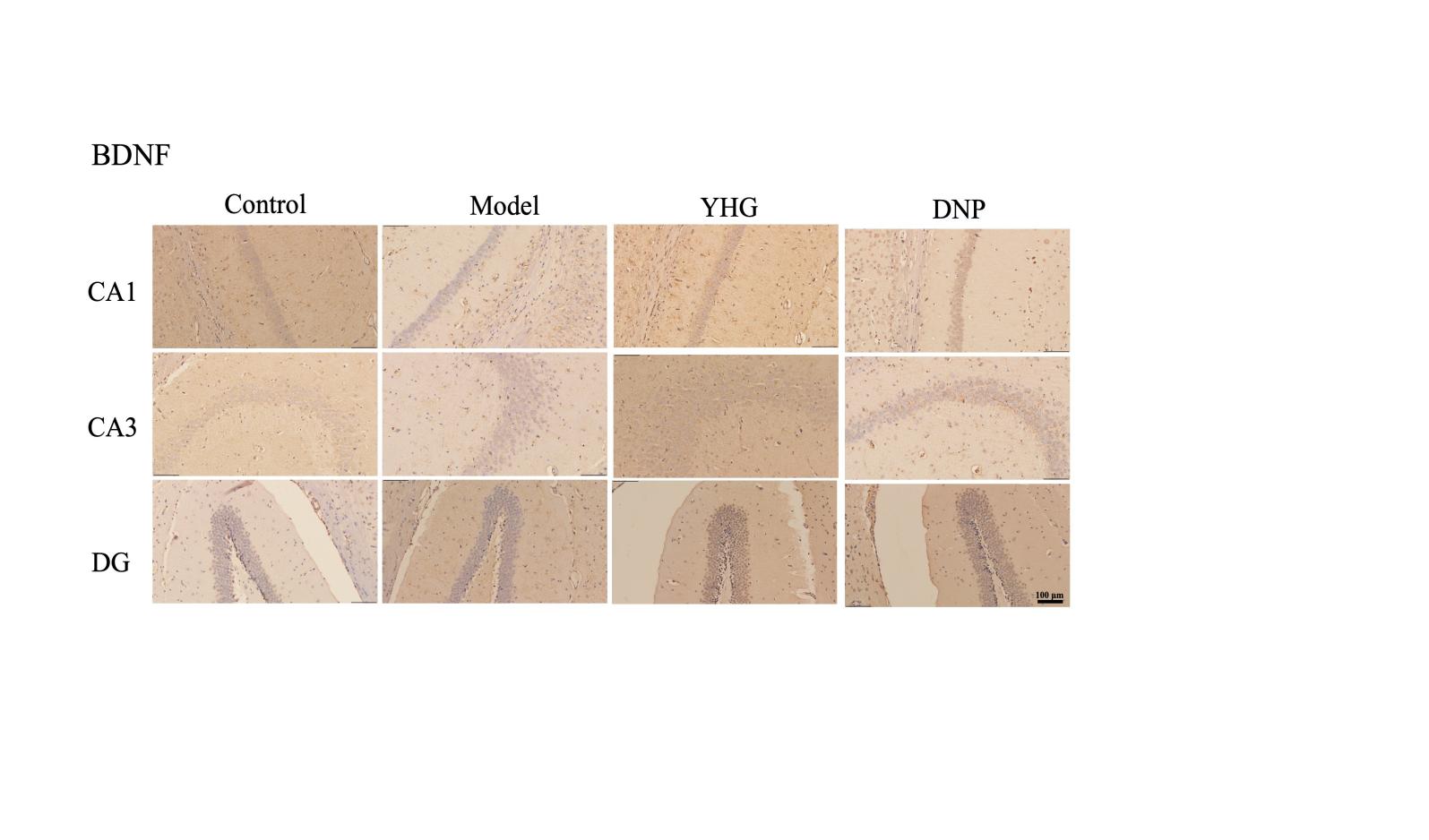


**Supplementary Figure 1.** Immunohistochemical staining of BDNF (Repeat 1)


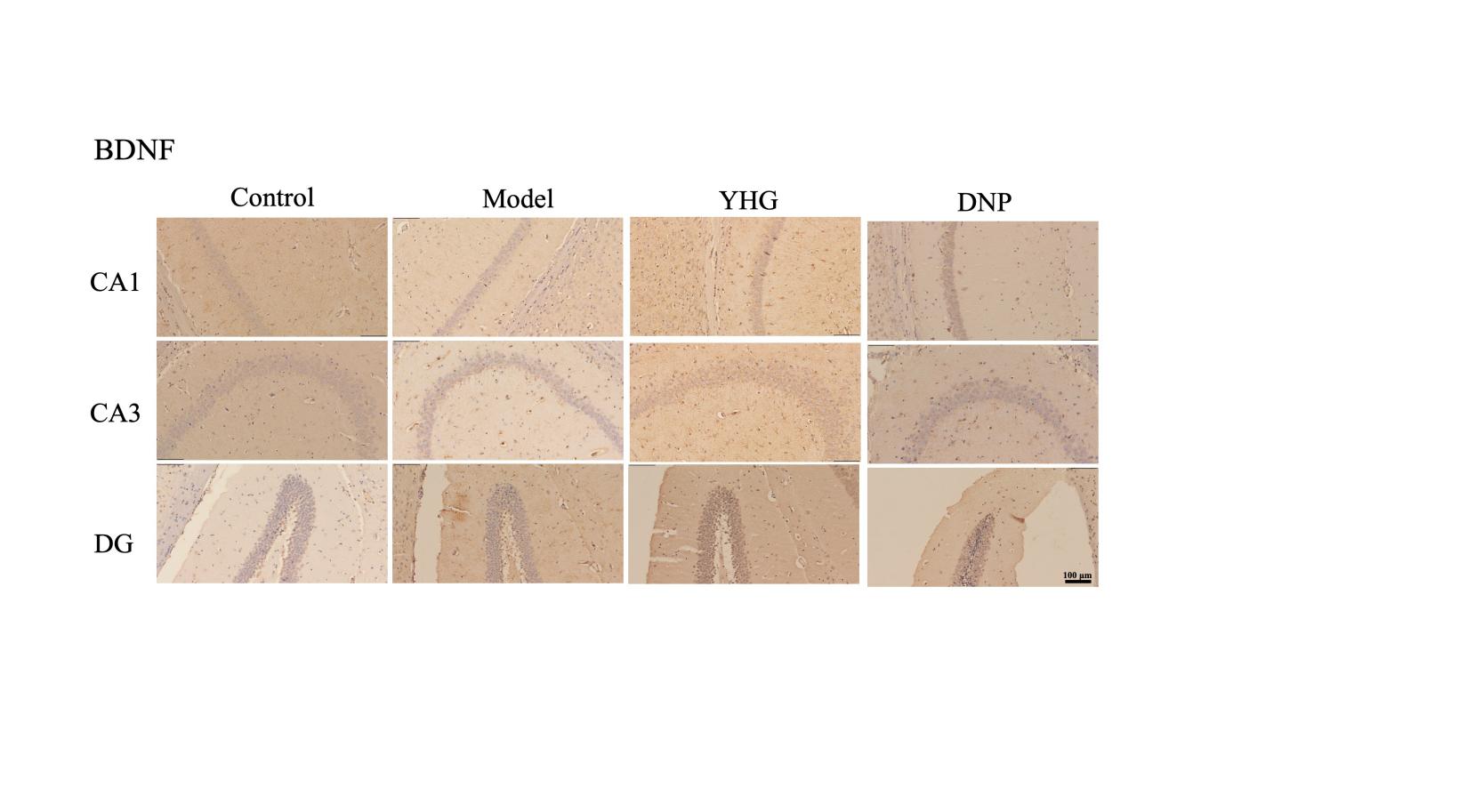


**Supplementary Figure 2.** Immunohistochemical staining of BDNF (Repeat 2)


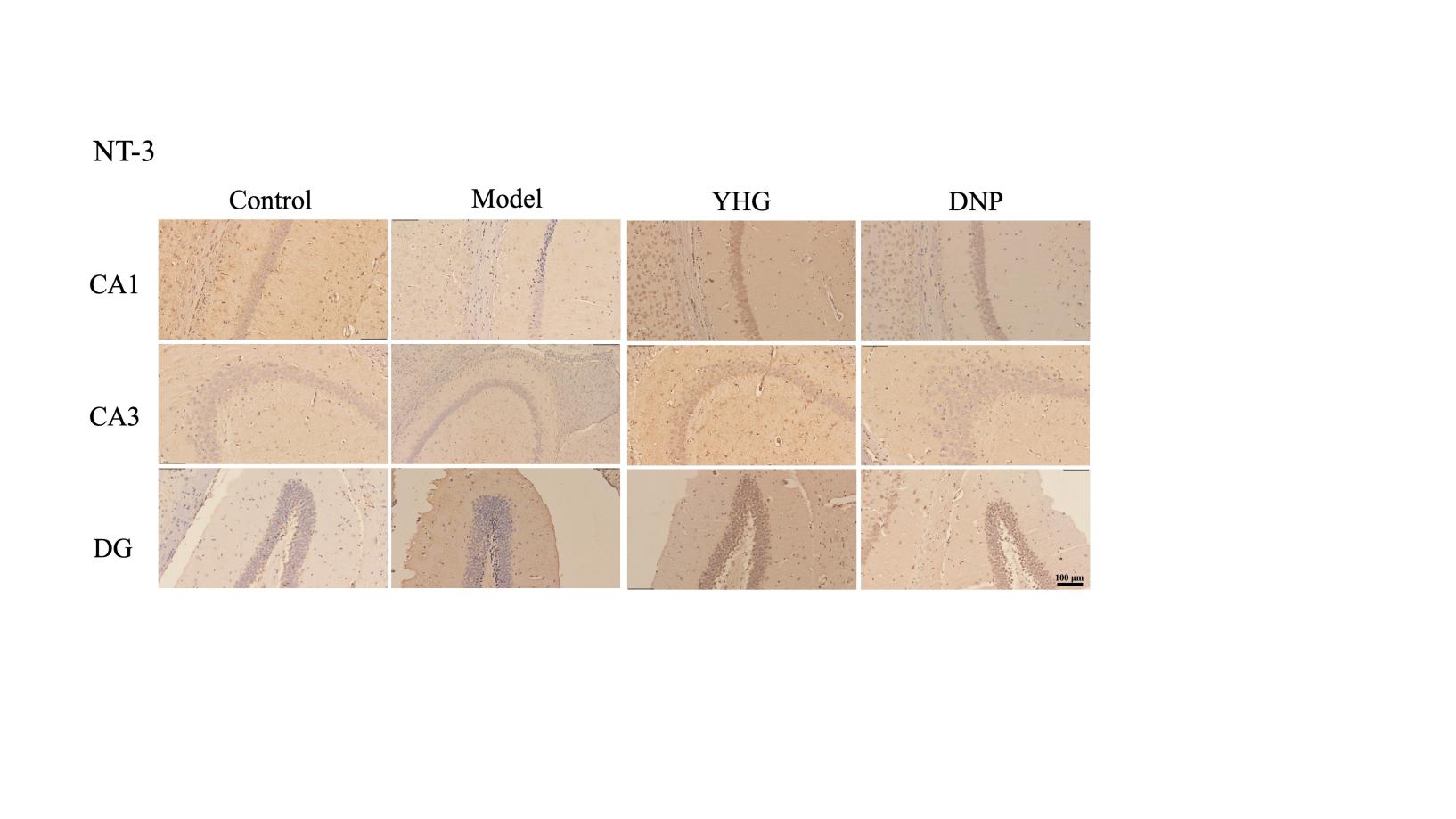


**Supplementary Figure 3.** Immunohistochemical staining of NT-3 (Repeat 1)


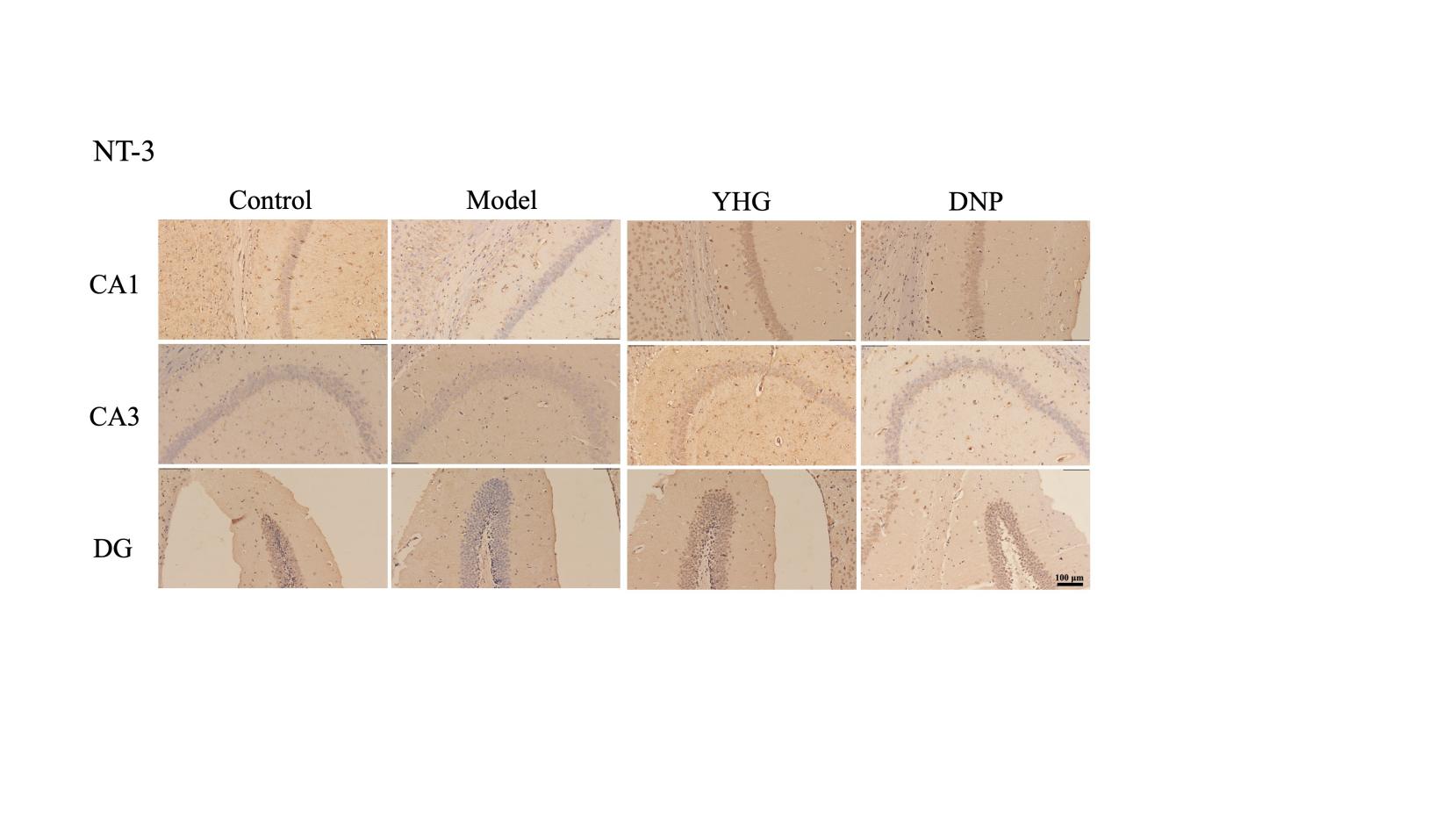


**Supplementary Figure 4.** Immunohistochemical staining of NT-3 (Repeat 2)


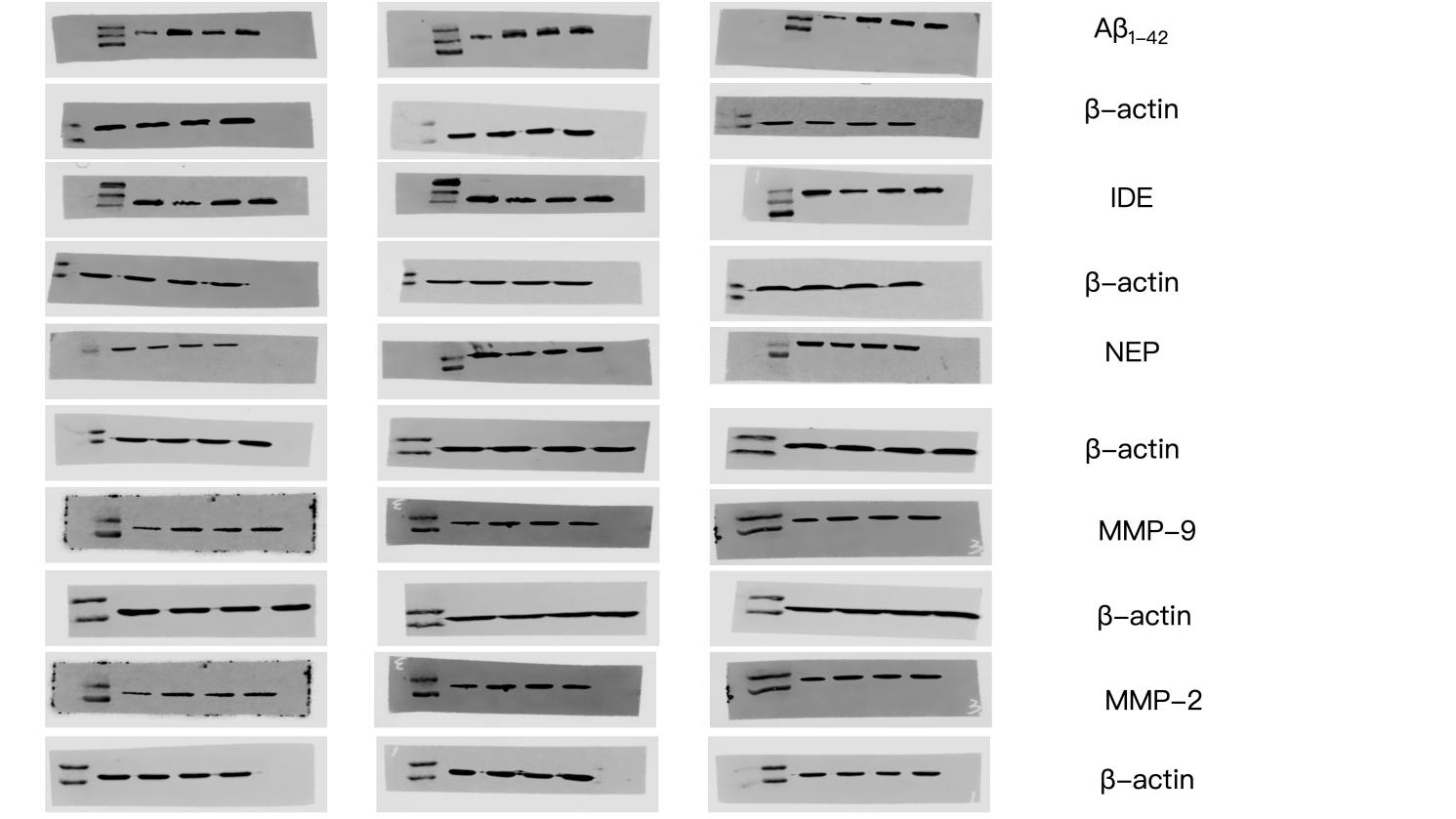


**Supplementary Figure 5.** Expression levels of β-actin、Aβ_1-42_、IDE、NEP、MMP-9、MMP-2 in different groups(n=3)
